# Supplementary material for: Health Literacy in People with Type 1 Diabetes: A Scoping Review
Source: Int J Environ Res Public Health. 2025 May 31;22(6):869. doi: 10.3390/ijerph22060869 (PMC12192628; doi:10.3390/ijerph22060869)
Supplement: Supplementary file 1 [file ijerph-22-00869-s001.zip › Supplementary File S1.pdf]

Supplementary File S1. Complete search strategy

Database: PubMed

(((("Diabetes Mellitus, Type 1"[Mesh]) OR (((((((((((((((((((IDDM[Title/Abstract]) OR T1DM[Title/Abstract]) OR "Type 1 Diabetes"[Title/Abstract]) OR "Autoimmune Diabetes"[Title/Abstract]) OR "Juvenile Onset Diabetes"[Title/Abstract]) OR "Juvenile-Onset Diabetes"[Title/Abstract]) OR "Brittle Diabetes Mellitus"[Title/Abstract]) OR "brittle diabetes"[Title/Abstract]) OR "diabetes mellitus type 1"[Title/Abstract]) OR "diabetes mellitus type I"[Title/Abstract]) OR "diabetes type 1"[Title/Abstract]) OR "diabetes type I"[Title/Abstract]) OR "early onset diabetes mellitus"[Title/Abstract]) OR "insulin dependent diabetes"[Title/Abstract]) OR "juvenile diabetes"[Title/Abstract]) OR "juvenile diabetes mellitus"[Title/Abstract]) OR "type I diabetes"[Title/Abstract]) OR "type I diabetes mellitus"[Title/Abstract]) OR "Insulin Dependent Diabetes Mellitus"[Title/Abstract]) OR "Insulin-Dependent Diabetes Mellitus"[Title/Abstract])))) AND (health literacy[mesh] OR (health[ti] AND literacy[ti]) OR ("health literacy" OR "health literate" OR "medical literacy") OR (functional[tw] AND health[tw] AND literacy[tw]) OR numeracy OR ((low literate[ti] OR low literacy[ti] OR literacy[ti] OR illiteracy[ti] OR literate[ti] OR illiterate[ti] OR reading[mh] OR comprehension[mh] OR "information literacy"[mesh]) AND (health promotion[major] OR health education[major] OR patient education[major] OR Communication Barriers[major] OR communication[major:noexp] OR health knowledge,attitudes,practice[major] OR attitude to health[major])) OR (comprehension[major] AND educational status[major]) OR (family[ti] AND literacy[ti]) OR (("drug labeling" OR Prescriptions [mh]) AND ("comprehension" OR "numeracy")) OR "low health literacy"[tw] OR "ehealth literacy"[tw] OR "limited health literacy"[tw] OR "low numeracy"[tw] OR ((cancer[ti] OR diabetes[ti]) AND (literacy[ti] OR comprehension[ti])) OR "adult literacy" OR "limited literacy" OR "patient understanding"[ti] OR "disease knowledge"[tw] OR ((self care [major] "self care"[tw] OR "self-care"[tw]) AND perception[mh]) OR (comprehension AND food labeling[mh]) OR (comprehension AND informed consent) OR (comprehension AND insurance, health))

Results: 243 (03-01-2025)

Database: Embase

('diabetes mellitus, type 1'/exp OR iddm:ti,ab OR t1dm:ti,ab OR 'type 1 diabetes':ti,ab OR 'autoimmune diabetes':ti,ab OR 'juvenile onset diabetes':ti,ab OR 'juvenile-onset diabetes':ti,ab OR 'brittle diabetes mellitus':ti,ab OR 'brittle diabetes':ti,ab OR 'diabetes mellitus type 1':ti,ab OR 'diabetes mellitus type i':ti,ab OR 'diabetes type 1':ti,ab OR 'diabetes type i':ti,ab OR 'early onset diabetes mellitus':ti,ab OR 'insulin dependent diabetes':ti,ab OR 'juvenile diabetes':ti,ab OR 'juvenile diabetes mellitus':ti,ab OR 'type i diabetes':ti,ab OR 'type i diabetes mellitus':ti,ab OR 'insulin dependent diabetes mellitus':ti,ab OR 'insulin-dependent diabetes mellitus':ti,ab) AND ('health literacy'/exp OR (health:ti AND literacy:ti) OR 'health literacy' OR 'health literate' OR 'medical literacy' OR (functional AND health AND literacy) OR numeracy OR 'low literate':ti OR 'low literacy':ti OR literacy:ti OR illiteracy:ti OR literate:ti OR illiterate:ti OR 'reading'/exp OR 'comprehension'/exp OR 'information literacy'/exp)

Results: 746 (03-01-2025)

Database: CINAHL

ALL ((('diabetes AND mellitus, AND type AND 1') OR ("t1dm :ti,ab") OR ("'type 1 diabetes':ti,ab") OR ('autoimmune AND diabetes':ti,ab) OR ('juvenile AND onset AND diabetes':ti,ab) OR ('juvenile-onset AND diabetes':ti,ab) OR ('brittle AND diabetes AND mellitus':ti,ab) OR ('brittle AND diabetes':ti,ab) OR ('diabetes AND mellitus AND type AND 1':ti,ab) OR ('diabetes AND type AND 1':ti,ab) OR ('diabetes AND type AND i':ti,ab) OR ('early AND onset AND diabetes AND mellitus':ti,ab) OR ('insulin AND dependent AND diabetes':ti,ab) OR ('juvenile AND diabetes':ti,ab) OR ('juvenile AND diabetes AND mellitus':ti,ab) OR ('type AND i AND diabetes':ti,ab) OR ('type AND i AND diabetes AND mellitus':ti,ab) OR ('insulin AND dependent AND diabetes AND mellitus':ti,ab) OR ('insulin-dependent AND diabetes AND mellitus':ti,ab)) AND ('health AND literacy'))

Results: 17 (03-01-2025)

Database: Scopus

ALL ((('diabetes AND mellitus, AND type AND 1') OR ("t1dm" :ti,ab) OR ('type 1 diabetes':ti,ab) OR ('autoimmune AND diabetes':ti,ab) OR ('juvenile AND onset AND diabetes':ti,ab) OR ('juvenile-onset AND diabetes':ti,ab) OR ('brittle AND diabetes AND mellitus':ti,ab) OR ('brittle AND diabetes':ti,ab) OR ('diabetes AND mellitus AND type AND 1':ti,ab) OR ('diabetes AND type AND 1':ti,ab) OR ('diabetes AND type AND i':ti,ab) OR ('early AND onset AND diabetes AND mellitus':ti,ab) OR ('insulin AND dependent AND diabetes':ti,ab) OR ('juvenile AND diabetes':ti,ab) OR ('juvenile AND diabetes AND mellitus':ti,ab) OR ('type AND i AND diabetes':ti,ab) OR ('type AND i AND diabetes AND mellitus':ti,ab) OR ('insulin AND dependent AND diabetes AND mellitus':ti,ab) OR ('insulin-dependent AND diabetes AND mellitus':ti,ab)) AND ('health literacy':ti,ab))

Results: 14 (03-01-2025)

Database: Web of Science

ALL= ((('diabetes mellitus, type 1') OR ('diabetes AND type AND 1':ti,ab) OR ('diabetes AND type AND i':ti,ab) OR ('early AND onset AND diabetes AND mellitus':ti,ab) OR ('insulin AND dependent AND diabetes':ti,ab) OR ('juvenile AND diabetes':ti,ab) OR ('juvenile AND diabetes AND mellitus':ti,ab) OR ('type AND i AND diabetes':ti,ab) OR ('type AND i AND diabetes AND mellitus':ti,ab) OR ('insulin AND dependent AND diabetes AND mellitus':ti,ab) OR ('insulin-dependent AND diabetes AND mellitus':ti,ab)) AND ('health AND literacy'))

Results: 289 (03-01-2025)

Database: Google Scholar

intitle:"type 1 diabetes" intitle:"health literacy"

Results: 40 (03-01-2025)
