# Supplementary material for: Health Literacy in People with Type 1 Diabetes: A Scoping Review
Source: Int J Environ Res Public Health. 2025 May 31;22(6):869. doi: 10.3390/ijerph22060869 (PMC12192628; doi:10.3390/ijerph22060869)
Supplement: Supplementary file 1 [file ijerph-22-00869-s001.zip › Supplementary File S2.pdf]

Supplementary File S2. Synthesis of results

First author, year of publication and country	Knowledge synthesis method/Study design	Aim	Research focus	Population/Sample characteristics	Barriers and facilitators for health literacy	Key findings and outcomes
Abrams C., 2023 USA	Observational study	To evaluate nutritional knowledge and perceived illness burden in young adults with T1DM.	Nutrition literacy.	N=42 participants completed survey. Age 18-30 years.	Not reported.	The burden of diabetes frequently prompts people to miss social engagements owing to food worries. Increased burden is often due to the difficulties of disease management rather than a lack of dietary understanding. Those who understand carbohydrate counting are more likely to avoid eating when their blood sugar levels are abnormal, as opposed to those who do not.
Alderson P., 2005 UK	Qualitative study	To explore the attitudes of children with T1DM regarding their illness.	Children's perspectives and ambitions, and their abilities to participate in the daily care.	n=15 children aged 3–6 and 10–12 years + n=9 children aged 6–11 years	Not reported.	Children around 4 years old can grasp diabetes control basics, enabling informed decisions for better health. Experience, not age, develops the capacity to consent. Their involvement is crucial for

Supplementary File S2. (*cont.*)

						managing the condition.
Alruhaim H.Y., 2021 Saudi Arabia	Observational study	To assess the level of diabetes numeracy in Saudi adults with insulin-treated diabetes and its correlation with diabetes self-management.	Diabetes numeracy and self-management.	The sample included 279 patients with either T1DM or T2DM. Most were female (58.8%), with 63% under the age of 30. Type 1 diabetes accounted for 72.8%, with 67% exclusively using insulin. Around 44% had been diagnosed for over 10 years, with an average A1c level of 9.2%.	Not reported.	Most patients (81.7%) scored below 50% on the DKS, indicating a lower level of diabetes numeracy, which correlated with decreased self-management behaviors.
Arghittu A., 2022 Italia	Observational study	To explore the relationship between nutritional knowledge, attitudes, and behaviors among diabetic patients, encompassing their dietary habits and adoption of healthy lifestyles, even amidst lockdown conditions.	Knowledge of foods and healthy lifestyles.	The study involved 321 individuals diagnosed with either Type 1 or Type 2 diabetes. Among them, 52% were female and 48% were male, with mean ages of 61.1 ± 18.5 years and 62.0 ± 15.1 years, respectively. No significant differences were observed between the genders.	Facilitators: Follow-up significantly improves knowledge levels, while self-management educational interventions seem to slightly decrease patient knowledge levels, although not significantly.	211 out of 256 respondents (82.4%) accurately identified the definition of diabetes. Females demonstrated significantly higher knowledge of food and nutrition compared to males, with knowledge levels decreasing as patient age increases.
Barnard K.D., 2014 UK	Conference proceeding (Observational study)	To assess the comprehension of alcohol and carbohydrate content in commonly consumed beverages among young adults with T1DM.	Knowledge of alcohol.	N=547 participants aged 18–30 (mean age 24.5 ± 3.7 years) took part: 341 women and 192 men. Among them, 365 (66.7%) consumed alcohol, with 142 females (41.6%) and 83 males (43.2%) scoring above the ≥ AUDIT3 cut-off, suggesting potential problem drinking.	Not reported.	Knowledge accuracy regarding alcohol units was low, with only 7.3% (n = 40) achieving ≥ 5 correct answers out of ten. Similarly, understanding of carbohydrate content was inadequate, as no participants scored

Supplementary File S2. (*cont.*)

						≥ 5 correct answers out of ten.
Barnard K.D., 2014 UK	Conference proceeding (Observational study)	To evaluate young adults with T1DM comprehension of alcohol and carbohydrate content in commonly consumed beverages.	Knowledge of alcohol.	In the study involving 547 participants aged 18-30 years, with a mean age of 24.5 years, 66.7% reported alcohol consumption. Notably, 32.9% of female participants and 22.6% of male participants scored above the AUDIT-C cut-off for increased risk drinking.	Not reported.	Participants' knowledge of alcohol units and carbohydrate content was notably lacking. Only 7.3% (n = 40) correctly identified the alcohol content of ≥6 out of 10 drinks, while none correctly identified the carbohydrate content of ≥6 out of 10 drinks.
Bejani J., 2024 Iran	Semi-experimental study	To evaluate the impact of a literacy promotion training program based on social learning theory on the self-efficacy and social anxiety of adolescents with T1DM.	HL, self-efficacy and social anxiety.	N= 66 adolescents aged 15–18 years with T1DM	Facilitators: healthcare professionals who provide information and training tailored to the individual's level of health literacy achieve better understanding and greater effectiveness.	HL training is effective in improving self-efficacy and reducing social anxiety among adolescents with T1DM.
Bouclaous C., 2022 Lebanon	Observational study	To assess numeracy skills in Lebanese individuals with diabetes.	Numeracy skills.	N=299 participants with a mean age of 47.4 ± 19.8 years, 52% were woman. N=101 have T1DM	Not reported.	62% of participants with diabetes demonstrated inadequate numeracy skills. T1DM generally showed better numeracy compared to those with T2DM. Furthermore, there was a correlation between higher numeracy skills and controlled A1c levels.

Supplementary File S2. (*cont.*)

Briggs Early K., 2023 USA	Editorial	To evaluate carbohydrate counting knowledge	Carbohydrate counting.	1 study mentioned (n= 224 adult patients (mean age 28.2 years) who were able to read Arabic and had a diagnosis of T1DM for at least one year.	Facilitators: repeated education sessions, rather than a one-time approach, were associated with higher knowledge levels, reinforcing the need for continuous learning. Technology has been proposed to support carbohydrate counting, its effectiveness in improving accuracy is still limited.	Participants with higher scores on the AdultCarbQuiz showed better glycaemic control, confirming the importance of carbohydrate counting education in diabetes management. Repeated education sessions, rather than a one-time approach, were associated with higher knowledge levels, reinforcing the need for continuous learning.
Broos B., 2020 Belgium	Conference proceeding (Observational study)	To determine if diabetes knowledge and health literacy would affect glucose control outcomes following one year of CGM usage among adults with T1DM	CGM use.	N=857 patients, age 46–15 years; diabetes duration: 24–14 years	Not reported.	No associations were observed between diabetes knowledge/health literacy and glycemic control after one year of CGM use.

Supplementary File S2. (*cont.*)

Broos B., 2021 Belgium	Observational study	To explore the potential influence of diabetes knowledge and health literacy on glycemic control following one year of intermittently scanned CGM.	CGM use.	The study involved 851 individuals diagnosed with T1DM, with an average age of 45.4 years and a diabetes duration of 25.1 years. The majority were male (53%), with a mean baseline HbA1c of 7.9%.	Not reported.	Diabetes knowledge and health literacy would influence glycemic control, severe hypoglycemia, hypoglycemic coma, and days of work or school absenteeism in people with T1DM starting with CGM during 1 year of routine clinical follow-up.
Campos Barrera E., 2011 Mexico	Conference proceeding (Observational study)	To assess the effect of diabetes-related numeracy, self-care activities on treatment goals in individuals with T1DM.	Diabetes numeracy, self-care activities.	N=50 people with T1DM participated, comprising of 31 women and an average diabetes duration of 10.5 years (1-49). The 19 men. They average A1c level was 8.86% (5.50-15.33) and patients checked their blood glucose levels 4 times a day (1-7).	Not reported.	Low diabetes self-care and numeracy scores were linked to higher A1c levels. Poor glycemic control was associated with low diabetes-related numeracy and self-care levels.
Chaytor N. S., 2015 USA	Observational study	To evaluate factors associated with severe hypoglycaemia in older adults (≥ 60 years old) with longstanding T1D (≥ 20 years)	Numeracy and hypoglicaemia	n= 201 participants. Median (interquartile range) age at enrolment was 66 (63–71) years, range: 60–86	Barriers: older age, lower education, higer depression symptoms are associated with problems with the complex mental operations. Facilitators: early identification of risk factors.	The overall severity of cognitive deficits was independently associated with both diabetes numeracy and Instrumental Activities of Daily Living, after controlling for age, education, frailty, and depression.
Cheng L.J., 2019 Singapore	Systematic review	To explore factors that can be addressed to promote good glycemic control in patients with diabetes	Glycemic control	24 articles (6 studies are focused on the aim of this research)	Facilitators: higher socioeconomic status and better diabetes-related knowledge are	Poor diabetes-related knowledge has been strongly associated with

					associated with improved glycemic control, facilitated by greater resource access and better adherence to self-management practices.	low educational levels and older age. Research indicates a significant relationship between inadequate diabetes knowledge and poor glycemic control.<br>Poor diabetes-related knowledge negatively impacts adherence to self-management practices, including medication compliance and blood glucose monitoring.
Chima C., 2020 USA	Systematic review	To assess a relationship between HL and its domains and medication engagement among adults with T1DM/T2DM	Medication engagement.	21 articles (1 study is focused on the aim of this research)	Not reported.	HL was associate with medical engagement but numeracy was not.

Supplementary File S2. (*cont.*)

De Charvalo Messora C.T., 2018 Brazil	Conference proceeding (Observational study)	To evaluate the levels of health literacy and DD in T1DM patients and the impact on adherence to treatment and glycemic control.	HL, DD and adherence to treatment.	N=90 diabetics patients type 1, 68.8% women, age 33.9 year ± 11.4; A1c 9% ± 2.1, T1DM time 19.2 years ± 9.3. 86.6% presented adequate literacy, 6.6% marginal and 6.6% inappropriate.	Not reported.	Most individuals with T1DM exhibit adequate health literacy and high diabetes distress scores. Non-adherence to treatment is associated with elevated diabetes-related distress (PAID), dyslipidemia, hypertension, unmarried status, retinopathy, and occupational status. These factors, with the exception of diabetes-related distress, contribute to an increased risk of worsening adherence to T1DM treatment.
De Morais Borges Marques R., 2018 Brazil	Conference proceeding (Observational study)	To evaluate the level of health and nutrition literacy among adults with type 1 diabetes and its correlation with treatment adherence, glycemic control, and nutritional status.	Health, nutrition literacy, diabetes management and nutritional status.	N= 47 participants, 66% were female with a mean age of 27 years (SD 0.04). 64% had a high school education and 66% had a monthly household income up to two minimum wages. Only 74.5% had access to sanitary sewage. The mean age at diagnosis was 10 years (SD 0.01). 40.4% of the participants reported complications due to diabetes.	Not reported.	Among the participants, 83% exhibited poor health and nutrition literacy. Factors such as gender, schooling, income, and anthropometric variables showed no association with literacy levels. Low HL was linked to poorer glycemic control.

Drown L., 2023 Malawi	Qualitative study	To investigate the effects of living with T1DM understanding of the disease, self-management practices, and factors facilitating or hindering T1DM care at two clinics in Neno, Malawi.	Experiences, knowledge and self-management.	N=23 participants: n=8 people with T1DM, mean age 31.8 y, 66.6% male + (n=4 family, n=9 providers, n=2 civil society member)	Facilitators: continued education results in improved diabetes self-management practices. Barriers: food insecurity, cost of food.	Newly diagnosed individuals with T1DM or those with low literacy levels may initially find it challenging to grasp self-management practices.
Esen I., 2018 Turkey	Observational study	To investigate the relationship between HL, diabetic control, and diabetic complications in patients with T1DM	HL, diabetic control and complication	N=106 (63 female and 43 male) patients with type 1 TDM with a mean age of 32 years were studied	Facilitators: physicians should consider the positive impact of education to improve disease management and prevent complications.	Low health literacy in patients with type 1 diabetes mellitus was linked to a higher prevalence of retinopathy.
Esen I., 2020 Turkey	Observational study	To investigate the relationship between HL and QoL in T1DM patients.	HL and QoL.	N=155 patients with T1DM.The mean duration of diabetes was 11 years. Diabetes complications were present in 46.45% of patients; 27.09% of the patients had other diseases	Not reported.	HL and QoL were found to be correlated with diabetes complications. Patients with low HL may encounter difficulties accessing treatment services when diabetes complications arise, potentially leading to a negative impact on their quality of life.
Eyüboğlu E., 2016 Turkey	Observational study	To assess the impact of HL and patient empowerment on diabetes self-care behaviour	HL and patient empowerment	n=302 patients with T1D or T2D. 65.3% were female. mean age was 51.4 years	Facilitators: empowerment plays a more significant role than health literacy in promoting habitual self-care behaviors in diabetes patients. Barriers: factors like medical necessity and	Health empowerment, particularly its impact dimension, drives self-care behaviors, while HL has a limited role, as patients already

Supplementary File S2. (*cont.*)

					perceived lack of choice can reduce the influence of empowerment on specific behaviors.	understand these behaviors. Empowerment focuses on self-management, whereas HL supports decision-making.
Evans E. I., 2024 USA	Observational study	To assess HL levels in T1D/T2D patients with CGM	HL	n=82 participants. N=21 (25.6%) have T1DM. Mean age 51.6 y	Facilitators: comprehensive patient education, the use of teach-back methods, and practical experience with CGMs can significantly enhance HL, empowering patients to better manage their diabetes through improved understanding of CGM data.<br>Barriers: limited income reduces access to educational resources, affecting HL growth.	CGM have been shown to improve clinical outcomes in diabetes, particularly in reducing A1c levels. Patients with lower HL, as measured by the HLS/SNS questionnaire, were more likely to have lower education levels, lower household income, and public insurance. Participants with higher HL scores reported greater understanding and comfort in using CGM.
Gandhi K., 2016 USA	Narrative review	To describe demographics, socioeconomic characteristics, acculturation and to assess the relationship with HL	Biopsychosocial factors.		Not reported.	Hispanics and Asians have lower HL than Whites in the US. People with limited English proficiency and low HL are most vulnerable to health problems.

Gomes M., 2020 Brazil	Observational study	The study aimed to identify factors influencing HL in diabetes patients and to evaluate HL's impact on glycemic control.	Educational level , glycemic control.	N=347 patients n=144 with T1DM and n=203 with T2DM. T1DM. Literate n=119,age 36.5 ± 12.3. illiterate n=25, age 42.2 ± 12.1.	Not reported.	T1DM patients with A1c <7.0% had adequate HL. T2DM patients had a higher prevalence of inadequate HL. 18% of young T1DM patients had inadequate HL. Age and years of school were important for better S-TOFHLA performance.
Hillson R., 2016 UK	Editorial	To highlight the importance of accessible, clearly written educational materials to support health literacy, improve diabetes self-care knowledge	HL and diabetes knowledge	People with T1DM/ T2DM	Not reported	A systematic review consistently linked low health literacy to poorer diabetes knowledge but found insufficient evidence of its independent impact on diabetes care processes or outcomes. Among adults with T1DM, poor literacy and numeracy skills were associated with higher HbA1c, particularly for those with lower numeracy, independent of confounders.
Itzkcovitz A., 2021 Canada	Observational study	To understand the level of nutrition and food literacy among young Canadian adults living with T1DM.	Food literacy.	n= 236 young adult with T1DM and n=191 control group. Patients with T1DM were slightly older than controls and more reported being Caucasian. 82% were	Not reported.	The proportion of young Canadian adults living with T1DM reporting adequate food literacy was greater than that among controls.

				female. The average duration of participants living with T1DM was 10.2 years. 60,5% used insulin pump and 63,7% used carbhoydrate counting for mealtime insulin adjustments.		Dietary knowledge, skills, confidence, and intake were higher in people with T1DM compared to controls.
Kane K., 2022 USA	Editorial	To assess the role of HL in management of T1DM.	T1DM management.	8 studies mentioned (1 study responded research question: n=120 Turkish adolescents with T1DM and n=190 Turkish adolescents without T1DM).	Not reported.	Adolescents with T1DM have limited HL and mean A1c decreased with improve HL. HL improved T1DM management.
Kerr D., 2010 UK	Editorial	To highlight the impact of low numeracy and educational levels on diabetes management,	Numeracy	Adult with T1DM	Facilitators: Education programs focused on numeracy and literacy improve self-efficacy and glycemic control in adults with diabetes, though the benefits diminish over time. A potential solution to overcome numeracy and literacy barriers involves designing devices and training materials that incorporate computer-based algorithms, interactive multimedia, and personalized interventions, considering users' numeracy, literacy, language, culture, and age.	Low numeracy in patients complicates risk perception, screening, medication adherence, and treatment assessment, increasing the risk of severe hypoglycemia. Lower educational levels are associated with a higher risk of premature death, primarily due to vascular disease.
Lappenschaar T., 2012 Netherlands	Conference proceeding (Observational study)	To evaluate carbohydrates knowledge and numeracy, how these skills influence glycaemic control.	Carbohydrates knowledge, numeracy and glycemic control.	N= 150 adolescents and young adults with T1DM as well as parents of a child with T1DM with an insulin pump and with	Not reported.	The correlation coefficient between carbohydrate knowledge and HbA1c was found to be moderate.

				adequate glycaemic control, A1c <8.5% (69 mmol/l) participated.		Poorer glycemic control was associated with moderate carbohydrate knowledge and HbA1c levels.
Leduc B., 2024 Haiti	Conference proceeding (Observational study)	To describe literacy and numeracy in Haitian youth with T1D	HL and numeracy	N= 85 patients (age 0-25 years). Median age at diagnosis and diabetes duration were 14.3 and 3.1 years, respectively	Not reported	Lower HbA1c was predicted by younger age (p=0.013), but not by gender, diabetes duration, numeracy score, mother's education or Patient Diabetes Self-Management Scale score.
Mangi A. A., 2018 Pakistan	Observational study	To evaluate the knowledge in people with diabetes.	Knowledge in diabetes.	N=2025 participants n=171 have T1DM.	Barrier: absence of services for the management of people affected by the condition.	The overall knowledge score was unsatisfactory. Data from tool KAP.
Marciano L., 2019 Svizzera	Meta-analisi	To assess the role of HL in diabetes- related knowledge, self-care, and glycaemic control.	Knowledge, self-care and glycaemic control.	61 articles (1 study is on T1DM, 16 studies are on T1DM/T2DM).	Not reported.	Health literacy plays a crucial role in glycemic control, as reflected in A1c levels, and is closely linked to diabetes knowledge, resulting in better self-management and overall health outcomes. Notably, studies utilizing perception-based measures tend to demonstrate a stronger effect on self-care activities.

Supplementary File S2. (*cont.*)

Marden S., 2010 UK	Conference proceeding (Observational study)	To assess numeracy and literacy skills in individuals with T1DM and their relationship with glycemic control.	Numeracy and literacy.	N=112 people with T1DM mean current age 43.8 +/- 12.5 years, 47% male, mean duration of diabetes 22.0 +/-13.2 years.	Not reported.	A considerable portion of participants demonstrated low literacy skills, particularly in numeracy, which was associated with glycemic control. Those with numeracy skills at level 2 achieved better A1c levels compared to those below level 2 in the Skills for Life Initial Assessments. Poor numeracy has shown to have economic, social, and psychological ramifications.
Marden S., 2012 UK	Observational study	To assess the numeracy and literacy skills of individuals with T1DM and determine whether there is a correlation with glycemic control, while considering factors such as diabetes duration, education, demographics, and socioeconomic status.	Numeracy and literacy skills.	N=112 people with T1DM mean current age 43.8 +/- 12.5 years, 47% male, mean duration of diabetes 22.0 +/-13.2 years.	Not reported.	Many participants showed low literacy and low numeracy skills, linked to glycemic control. Those with numeracy skills at level 2 had lower A1c levels than those below level 2 (Skills for Life Initial Assessments). Poor numeracy has economic, social, and psychological implications. The association between numeracy and A1c levels remained unaffected by

Supplementary File S2. (*cont.*)

						socioeconomic factors.
Moosa F.Y., 2011 Johannesburg	Observational study	To establish if there was an association between the level of mathematical skill and degree of metabolic control in people with T1DM.	Mathematical skill and metabolic control.	In a sample of 53 children with type 1 diabetes, most fell within the age group of 12-15 years (43.40%); comprising 32 females (60.38%) and 21 males (39.62%), with 37 primary caregivers. Samples were not matched, and children over 18 years could provide their own consent. The mean age of the children was 12.92 ± 2.96 years (range 8-19 years). The mean A1c level for diabetic children was 12.84 ± 3.04%.	Not reported.	Patients and caregivers exhibited poor performance on basic math tasks, indicating a deficiency in math education. Low numeracy skills adversely affect diabetes care, as evidenced by a negative correlation between A1c levels and applied math scores.
Muniz L.H., 2019 Brazil	Conference proceeding (Observational study)	To determine which factors may influence HL in patients with T1DM and the influence of HL on glycemic control.	HL and glycemic control	N= 347 patients (n=144 with T1DM and n=203 with T2DM)	Facilitators: diabetes management should integrate health literacy evaluation with psychological and social considerations.	Many patients with T1DM or T2DM lacked adequate HL. T1DM patients generally performed better. Age and years of schooling were key factors influencing HL level. T1DM patients who self-identified as White, had more schooling, and adequate HL were more likely to achieve proper glycemic control.

Supplementary File S2. (*cont.*)

Naoko Naef A., 2023 Germany	Conference proceeding (Observational study)	To assess what potential distal technologies have for promoting HL in adolescent eith T1DM	Distal technologies.	n=12 medical doctors and n=20 adolescent with T1DM	Not reported	HL and empowerment were enhanced by peer support and trusting interactions with health professionals.
Olesen K., 2017 Denmark	Observational study	To investigate the association between each of the nine domains of the Health Literacy Questionnaire (HLQ) and HbA1c level in a large population of people with type 1 diabetes	Knowledge about health literacy and HbA1c among people with type 1 diabetes	N=1399 people with T1DM,half of respondents were women, and the majority had a tertiary education.	Not reported	Evaluating health info predicts diabetes management regardless of education or duration of diabetes. Higher health literacy lowers HbA1c, regardless of education.
Osborn C. Y., 2010 USA	Observational study	To describe the association between HL, numeracy, and diabetes self-efficacy.	HL, numeracy and self-efficacy.	N=383 people White or African American race,the mean age (SD) was 54 (13) years; 50% were female and 65% were White. 15% had T1DM.	Not reported.	31% of participants demonstrated health literacy below a ninth-grade level REALM, while 69% exhibited numeracy skills below this grade level WRAT-3 R. Increased diabetes self-efficacy correlates with lower A1c levels. When considering both, numeracy shows a stronger association with diabetes self-efficacy than HL.
Owusu B., 2023 Ghana	Qualitative study	To understand the management knowledge of T1DM among young individuals living with the condition in Ghana.	Knowledge and skills management.	Among the 47 participants, 28 were young people aged 14-24 self-managing T1DM, with equal representation of	Not reported	The findings revealed that young people with T1DM and their caregivers possessed

				males and females. The average age was 20, and the average duration of diabetes was 8 years. Among the participants, 15 had a family history of diabetes, and 19 were students living with primary caregivers. All young people with type 1 diabetes possessed an active National Health Insurance card. Among the 14 female participants, 3 had experienced unsuccessful pregnancies.		significant knowledge and skills in self-monitoring blood glucose and managing hyperglycemia. However, there were knowledge gaps regarding carbohydrate counting, managing hypoglycemia, and handling T1DM during significant life events like pregnancy.
Patrakeeva, E., 2013 Russia	Conference proceeding (Observational study)	To evaluate simply mathematical task and it's relation to quality on glicemic control	Numeracy	N= 36 patients with continuous subcutaneous insulin infusion.<br>2 groups:<br>1* frequent use of boluses per day;<br>2* less frequent usage of boluses	Not reported	A1c levels were comparable between groups. The Group 1 achieved higher scores on the test. Numeracy assessment was an important tool for successfully educating patients in continuous subcutaneous insulin infusion.
Piatt G., 2014 USA	Observational study	To determine the levels of FHL and if associations exist between level of FHL and levels of glycemic control.	Level of FHL and level of glycemic	N=70 African American people with T1DM (9.2%) or T2DM (90.8%); average age was 58.7 ± 13.5 years with 26.9% over age 65. Female (59.1%), insured (94.3%), and employed or retired (87.1%). Average diabetes duration was 18.3 ± 9.3 years.	Not reported	FHL assessment among insulin-treated African Americans with diabetes reveals common low diabetes-related FHL skills. These are associated with age, retirement status, and marginally

						with gender, but not with education level. Since income level correlates closely with education level, the lack of association between FHL and education suggests a similar absence of correlation between FHL and income level.
Rahimi Hassanabad V., 2023 Iran	Conference proceeding (Observational study)	To determine the relationship between HL, functional self-efficacy and social anxiety of adolescents with T1DM	HL, functional self-efficacy and social anxiety	N=60 adolescents. Highest frequency of adolescents with T1D was 15 years (46.7%) and (51.7% boys and 48.3% girls).	Not reported	The mean score of health literacy, self-efficacy and social anxiety was 48.99 ± 6.69 (insufficient), 123.78 ± 1.25 (Moderate) and 98.45 ± 4.9 (high), respectively. A direct and significant relationship was found between health literacy and self-efficacy ($P < 0.05$), an inverse and significant relationship was found between health literacy and social anxiety ($P < 0.05$)

Supplementary File S2. (*cont.*)

Reagan L., 2015 USA	Observational study	To examine relationships of self-care behavior, illness representation and diabetes knowledge.	Self-care behaviour.	N=124 individuals from 5 medium to high security state prisons. n=16 have T1DM. The sample was predominantly male, black or non-Hispanic white. More than half of the participants had adequate HL, the majority had a high school diploma, and 65% had a history of alcohol or drug abuse. Participants were relatively young at diagnosis, the majority were using insulin and had had diabetes for more than 5 years. 66% of participants had A1C levels ≥ of 8.0%.	Not reported.	Higher diabetes knowledge was associated with lower variance in A1C in linear regression analysis. However, this was not significant in the final multivariate regression model.
Röhling M., 2023 Germany	Observational study	To assess the HL regarding T1DM in childhood and adolescence.	HL	In 2007, n = 4383 and in 2019, n = 572 students from grades 5-12 (Lower level = grades 5–7; intermediate level = grades 8–10; upper level = ≥ grade 11) participated in the student surveys. A complete dataset was available for n = 4283 (97.7%) in 2007 and n = 485 (84.8%) in 2019. Lower level: 40.4% (2007), 25.2% (2019) Intermediate level: 49.0% (2007), 53.8% (2019) Upper level: 10.6% (2007), 21.0% (2019)	Not reported	No changes in HL related to diabetes and other cardiovascular risk factors were observed among 5th–12th grade students over the past 12 years.

Supplementary File S2. (*cont.*)

Sancrainte L., 2023 USA	Conference proceeding (Observational study)	To evaluate the level of HL and numeracy in adolescent patients aged 13-18 years with T1DM	HL and numeracy	N=23 patients (aged 13-18) living with T1DM	Not reported	Most participants (69.5%) had adequate HL and numeracy, while 26% showed low HL and numeracy, with scores increasing with age in youths. No correlation was found between HL and numeracy scores and HbA1c levels in caregivers or patients.
Sarkar U., 2017 USA	Letters	To discuss the impact of numeracy on diabetes.	Numeracy.	Not reported.	Not reported.	Patients with lower numeracy may have had higher rates of hypoglycemic episodes
Schwennesen N., 2018, Denmark	Observational study	To compare HL and self-care among visually impaired and sighted people with T1DM	HL and self-care	Compares two groups: visually impaired (n=38) and sighted (n=1387). The mean age is significantly higher in the visually impaired group (63 years vs 53 years, p<0.001).	Facilitators: educational, informational, and technological improvements are needed to enhance self-care and HL among visually impaired individuals with T1DM. Health professionals need training to support visually impaired individuals and access to non-visual self-monitoring tools for patient use.	HL shows no significant differences across most domains, except for finding and understanding health information to manage health, which are significantly better in the sighted group. Other aspects, including support, health management, and engagement with healthcare providers, show no significant group differences.
Seger M., 2024, Australia	Editorial	To emphasize that HL is crucial for the maintenance of individuals' health	HL	People with T1DM	Barriers: Inattention or inertia, which may occur when information is conflicting, complex, or overwhelming.	T1D management requires advanced technologies, medications, and treatments,

Supplementary File S2. (*cont.*)

					Suboptimal mental and physical well-being, limited knowledge, skills, and support, as well as insufficient time and financial resources, further hinder effective self-management and decision-making	demanding adequate HL. Low HL is linked to poorer health outcomes, reduced adherence, and difficulty navigating healthcare, especially when compounded by impairments or limited technological skills. Screening tools can help identify low HL and address its impact.
Turrin K.B., 2019, USA	Observational study	To assess diabetes numeracy levels of patients on insulin pump therapy and explore the glycemic control and diabetes self-management behaviors of patients with different diabetes numeracy levels on insulin pump therapy	Diabetes numeracy and glycemic control and self-management in patients with insulin pump	n=72 participants were enrolled in the study. The average age was 52 ± 14.9 years, the average A1C was 7.7% ± 1.2% (61 mmol/mol), the duration of diabetes was 27.3 ± 14.9 years, and the duration of insulin pump use was 3.4 ± 1.3 years. Forty-two participants (58.3%) were female	Barriers: lower Digital Numeracy (DN) scores were associated with older age, poorer self-management of diabetes, lower confidence in using insulin pump features, and higher A1C levels.	Many patients on insulin pump therapy struggle with diabetes numeracy, particularly older individuals and those with higher A1c levels. Notably, 19% of the patient population scored below 76%, indicating limited numeracy skills. This could significantly impact their ability to effectively manage insulin pump therapy.
Varela M., 2013 Portugal	Conference proceeding (Observational study)	To assess the relationship between HL and metabolic control in patients with T1DM.	Metabolic control.	N= 61 patients with T1DM.	Not reported	The HL influences significantly the metabolic control and that 44.3% of the diabetic patients had low levels of health literacy.

Supplementary File S2. (*cont.*)

Wagner S., 2023 Denmark	Observational study	To explore (1) the mediating role of social support and FHL in the relationship between socio-economic status and diabetes self-management in T1DM, (2) whether social support contributes to functional health literacy and (3) whether social support and functional health literacy independently contribute to diabetes self-management.	HL in health disparities	N=1396 participants with T1DM, 50.2% (n=595) males and 49.8% (n=591) females. The mean age was 55.8 years, and the mean A1c level was 60.4 mmol/mol. The mean A1c was higher than recommended values, and 75% (n=887) of respondents had an average A1c level above the recommended 53 mmol/mol. Most respondents had medium or high education, and participants generally scored high on the HLQ subscales.	Not reported	There was evidence of complementary or partial mediation of the effect of education on A1c, suggesting a role for FHL and social support. Social support increased FHL, the attributable improvement in A1c was very small but still statistically significant. The educational level had little effect on available social support.
Xu X.Y., 2018 Hong Kong	Systematic review	To investigate the relationship between HL and self-efficacy, and the relationships between the different subdomains of HL and self-efficacy	Self-efficacy	11 articles (1 study is focused on the aim of this research)	Not reported	There was a significant rela-tionship between HL and self-efficacy and numeracy and self-efficacy.
Zaugg S. D., 2014, USA	Observational study	To compare the diabetes numeracy and blood glucose control of patients who received care from diabetologists/endocrinologists in a diabetes-focused center to those receiving care from primary care physicians in primary care facilities and to evaluate the numeracy and blood glucose control of participating patients with T1DM versus those with T2DM	Diabetes numeracy in diverse cohorts of patients with diabetes across different healthcare settings	n=194 participants. The mean age was 53.08 years (SD = 15.14). The sample consisted of 39.2% male and 60.8% female participants.32% had T1D and 68% had T2D.	Barriers: low literacy and numeracy complicate self-care, adherence, and complex tasks like insulin dosing, often leading to errors or abandonment. Facilitators:screening and tailored education, combined with personalized tools and support, improve adherence and glucose control.	Diabetes numeracy predicts adherence to care but not glucose control. Patients treated by diabetes specialists showed higher numeracy, education levels, and completion of diabetes education compared to those treated by primary care physicians, yet these advantages did not

Supplementary File S2. (*cont.*)

						improve A1c levels.
Zuercher E., 2017 Switzerland	Observational study	To assess the association between FHL and self-reported process and outcome of care indicators in people with diabetes.	FHL	T0: N=519 non-institutionalised adults (≥18 years) with diabetes of at least one year duration, sufficient level of French, without cognitive impairment or gestational diabetes. Gender-60% male, age mean 65, 88% with T2DM. T1: N=381 participants with the same characteristics except for mean age at recruitment (more younger)	Not reported	Medical information shows that 52.5% have good FHL, 40.7% have moderate FHL and 6.8% have poor FHL. Low FHL is linked to lower income and longer diabetes duration. Physical activity recommendation increases as FHL decreases. Scores improve with increasing FHL. Care processes are not linked to FHL. Patients with intermediate FHL have lower self-efficacy.

Legend:
A1c- Glycosylated hemoglobin
AUDIT 3- Tool “Alcohol Use Disorders Identification Test”
CGM- Continuous Glucose Monitoring
DD- Diabetes-related Stress
DKS- Tool “Michigan diabetes knowledge scale”
FHL- Functional Health Literacy
HbA1c- Glycosylated haemoglobin
HLQ- Health Literacy Questionnaire
KAP- Knowledge Attitude and Practice
PAID- Problem Area in Diabetes
QoL- Quality of Life
REALM- Tool “Rapid Estimate of Adult Literacy in Medicine”
T1DM- Type 1 diabetes
T2DM- Type 2 diabetes
WRAT-3 R- Wide Range Achievement Test
